# ENhancing Assessment of Common Therapeutic factors (ENACT) tool: adaptation and psychometric properties in South Africa

**DOI:** 10.1017/gmh.2022.40

**Published:** 2022-08-12

**Authors:** Maxine Spedding, Brandon Kohrt, Bronwyn Myers, Dan J. Stein, Inge Petersen, Crick Lund, Katherine Sorsdahl

**Affiliations:** 1 Department of Psychology, University of Cape Town, Cape Town, South Africa; 2 Division of Global Mental Health, Department of Psychiatry and Behavioral Sciences, George Washington University, Washington, DC, USA; 3 Curtin enAble Institute, Faculty of Health Sciences, Curtin University, Western Australia, Australia; 4 Alcohol, Tobacco and Other Drug Research Unit, South African Medical Research Council, Cape Town, South Africa; 5 Department of Psychiatry and Mental Health, University of Cape Town, Cape Town, South Africa; 6 SAMRC Unit on Risk & Resilience in Mental Disorders, Dept of Psychiatry & Neuroscience Institute, University of Cape Town, Cape Town, South Africa; 7 Center for Rural Health, College of Health Sciences, University of KwaZulu Natal, Durban, South Africa; 8 Centre for Global Mental Health, Health Service and Population Research Department, Institute of Psychiatry, Psychology & Neuroscience, King's College London, London, United Kingdom; 9 Alan J Flisher Centre for Public Mental Health, Department of Psychiatry and Mental Health, University of Cape Town, Cape Town, South Africa

**Keywords:** Evidence-based psychosocial treatments, South Africa, therapist competencies

## Abstract

**Background:**

The ENhancing Assessment of Common Therapeutic factors (ENACT) tool measures a set of therapeutic competencies required for the effective psychological intervention, including delivery by non-specialists. This paper describes the systematic adaptation of the ENACT for the South African (SA) context and presents the tool's initial psychometric properties.

**Methods:**

We employed a four-step process: (1) Item generation: 204 therapeutic factors were generated by SA psychologists and drawn from the original ENACT as potential items; (2) Item relevance: SA therapists identified 96 items that were thematically coded according to their relationship to one another and were assigned to six domains; (3) Item utility: The ENACT-SA scale was piloted by rating recordings of psychological therapy sessions and stakeholder input; and (4) Psychometric properties: Internal consistency and inter-rater reliability of the final 12-item ENACT-SA were explored using Cronbach's alpha and intraclass correlation co-efficient (ICC) for both clinical psychologists and registered counsellors.

**Results:**

Although the original ENACT provided a framework for developing a tool for use in SA, several modifications were made to improve the applicability of the tool for the SA context, and optimise its adaptability other contexts. The adapted 12-item tool's internal consistency was good, while the inter-rater reliability was acceptable for both clinical psychologists and registered counsellors.

**Conclusion:**

The ENACT-SA is a reliable tool to assess common factors in psychological treatments. It is recommended that the tool be used in conjunction with assessment protocols and treatment-specific competency measures to fully assess implementation fidelity and potential mechanisms of therapeutic change.

## Introduction

The global mental health movement's drive to redress inequities in mental health care and reduce the treatment gap incorporates efforts to ensure that only effective and empirically-supported treatments are developed and employed (Kirmayer and Pedersen, 2014). Using evidence-based interventions (EBI) is essential, particularly in light of the widespread endorsement of the use of task-sharing models for delivering mental health interventions in poorly resourced settings (WHO, 2007). Support for task sharing has gained significant ground in low- and middle-income countries (LMIC), where the prevalence of untreated mental illness is high (Herman *et al*., 2009; Steel *et al*., 2014) and mental health resources are limited (Docrat *et al*., 2019).

Several systematic reviews and meta-analyses have shown that task sharing EBI for mental health to non-specialist health workers (NSHW), such as community health workers and trained peer providers, can effect therapeutic change (Clarke *et al*., 2013; Padmanathan and De Silva, 2013; Rahman *et al*., 2013; Singla *et al*., 2017). In South Africa, there is a small but growing body of literature to show that NSHWs, with training and ongoing supervision, are able to deliver a range of evidence-based therapies (Sorsdahl *et al*., 2015; Spedding *et al*., 2015; Myers *et al*., 2019; Calligaro *et al*., 2021; Petersen *et al*., 2021). While this evidence is growing, the research to date has significant limitations. Arguably, the most important limitation is a focus on outcomes, with limited attention given to understanding the factors that contribute to intervention effectiveness (van Ginneken *et al*., 2013; Selohilwe *et al*., 2019).

The factors that make psychotherapy effective have been debated at length (Leibert and Dunne-Bryant, 2015). While some researchers argue that treatment-specific factors are essential to therapeutic change; research of specialist-delivered psychotherapies has shown that no one model is more effective than another (Beutler *et al*., 2012; Fife *et al*., 2014). One study found that the unique contributions of model-specific techniques accounted for only 8% of the variance in outcomes (Wampold, 2015), while another meta-analysis found no evidence to show that specific psychotherapy factors resulted in positive therapeutic outcomes (Ahn and Wampold, 2001; Singla *et al*., 2017). As a result, support has increased for the notion of common therapeutic factors that promote positive therapeutic outcomes (Fife *et al*., 2014; Stamoulos *et al*., 2016).

Common therapeutic factors are defined as ‘variables that are common across psychotherapeutic modalities, that are unspecific to particular theories or techniques as defined by these unique modalities’ (Stamoulos *et al*., 2016). Theorists have proposed various models (Lundh, 2014) including both therapist or counsellor competencies (for example, ability to establish therapeutic alliance) and patient-related factors (for example, motivation to attend therapy) they consider common across the application of all major psychotherapeutic approaches, and which are critical for intervention effectiveness (Fife *et al*., 2014).

Although counsellor competencies are widely recognised as essential for therapeutic change, most task-sharing research has focused on the effectiveness of specific therapeutic modalities, emphasising therapy protocols and specific techniques with limited consideration given to understanding how therapist competencies influence outcomes. Recognising this gap, recent initiatives have highlighted the need to develop tools and guidelines for quantifying the competencies of non-specialist workers in delivering mental health interventions (Kohrt *et al*., 2020). Although several tools for expert ratings of therapeutic competence and quality are available, these have emerged from high-income countries (Margison *et al*., 2000; Cahill *et al*., 2008) and there are few initiatives that have explored these competencies objectively in LMICs where there is a larger reliance on NSHW (Kohrt *et al*., 2015a, 2018; Rahman *et al*., 2019; Restivo *et al*., 2020; Asher *et al*., 2021). For task-shared EBI to be disseminated and implemented widely in LMICs, scalable methods of assessing NSHW competence to deliver high-quality services are required (Kemp *et al*., 2019).

To this end, as part of the Programme to Improve Mental Health Care (PRIME) in Nepal, Kohrt *et al*. (2015a, 2015b) developed a tool for assessing counsellor competence among NSHW. The tool, ENhancing Assessment of Common Therapeutic factors (ENACT) (Kohrt *et al*., 2015b), measures a set of identified common factors: those therapeutic competencies and skills thought to be required for a counsellor to adequately deliver any EBI effectively. This tool was initially piloted in Nepal, Liberia, and Uganda (Kohrt *et al*., 2018; Jordans *et al*., 2021; Leichner *et al*., 2021). The ENACT has undergone a robust development process in low resource settings and has shown its utility in assessing the delivery of task-shared EBIs (Kohrt *et al*., 2020). Although there are examples of adapting the original ENACT tool by modifying existing items (Kohrt *et al*., 2020; Asher *et al*., 2021), there has not been a similar process that includes rating by local mental health professionals of the original pool of total items as well as local development of additional items.

This paper contributes to the growing body of research on psychological treatments in low resource settings by describing the systematic adaptation of the original ENACT for the South African context, known for its cultural diversity, and presents the initial psychometric properties of the ENACT-SA. The aim of the adaptation was to further develop the tool's responsiveness to context; to enhance its applicability to a range of formats, such as, training role plays, *in vivo* observations, or recorded sessions (both audio and video); to make it accessible for use by various stakeholders including, trainers, supervisors, peers/colleagues, and researchers; and to make it a suitable adjunct to support any task shared EBI for mental health.

## Methods

**Fig. 1.**
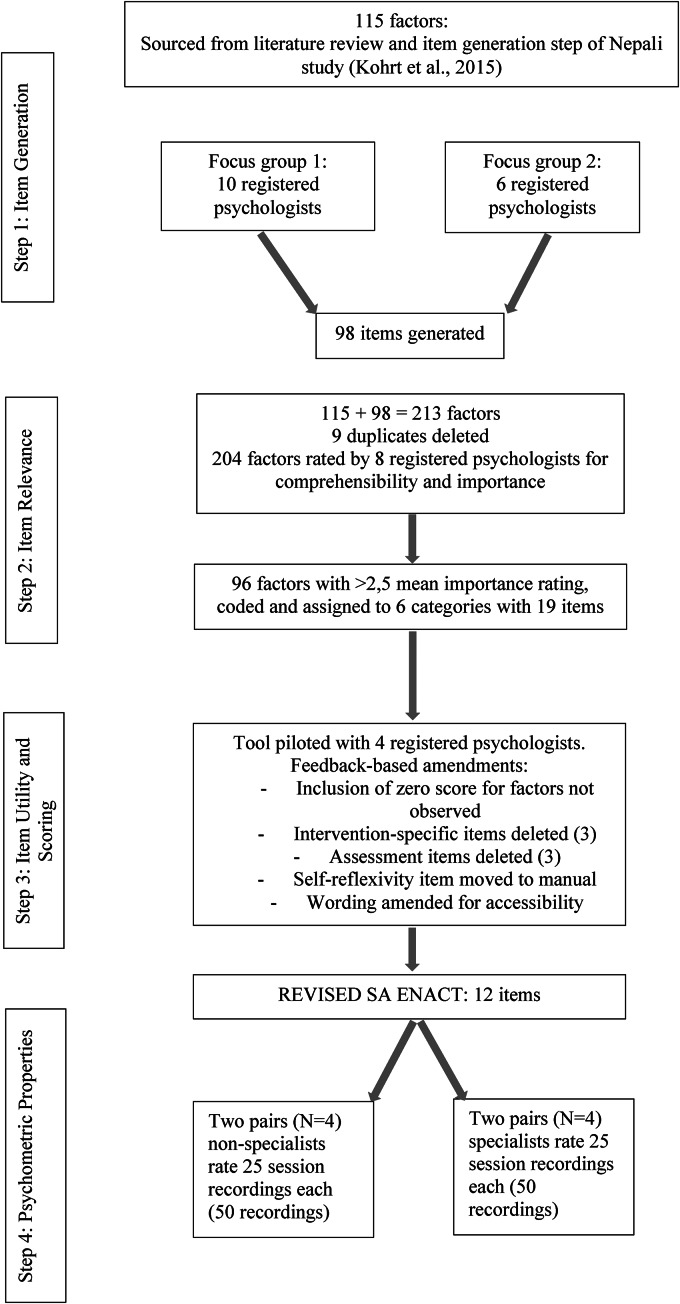
Procedures for systematic adaption of SA ENACT.

The methods and procedures employed in the adaptation of the ENACT to the South African context were largely drawn from those used to build the original ENACT (Kohrt *et al*., 2015a, 2015b) developed specifically for working with non-specialists in LMIC settings. The adaptation consisted of four steps: (1) generating items, (2) establishing item relevance, (3) assessing item utility and scoring, and (4) determining the adapted tool's psychometric properties (see Fig. 1). All steps in the South African adaptation were conducted with South African registered psychologists^†^^1^ (specialists) who have experience in public mental health settings, and who are familiar with task sharing approaches to mental health interventions. In the last step, four registered psychological counsellors (non-specialists) were also recruited to complete the psychometric properties assessment.

The session material used for this study were audio recordings of counselling sessions from Project MIND, a cluster randomised controlled trial (RCT) which has compared two approaches to integrating a new, task-shared counselling service for depression and unhealthy alcohol use into chronic disease care for HIV and diabetes in the Western Cape Province (described in (Myers *et al*., 2018b). In this service, facility-based counsellors (FBCs), a cadre of community health workers employed to provide health promotion and HIV adherence counselling services within PHC facilities, were trained to deliver a structured three-session blended motivational interviewing and problem-solving therapy (MI-PST) intervention. This intervention is described in detail elsewhere (Jacobs *et al*., 2020).

### Step 1: item generation

The original ENACT study generated a set of 115 potential items by reviewing the common factors competency assessment literature, which was dominated by high-income country research with mental health professionals, and conducting semi-structured discussion groups with Nepali therapists (Kohrt *et al*., 2015a). To adapt the tool for the South African context, two focus groups were conducted with a total of 16 psychologists to generate context-relevant items. Fifteen were registered clinical psychologists, while one was a registered counselling psychologist, with a mean of 13 years of registration in independent practice (range: 4–26 years). All had at least two years of experience in the public sector, while nine were employed in public health sector positions at the time. All were active and practicing psychotherapists. Focus group participants were shown the same four 15-minute video recordings of non-specialists role-playing counselling sessions in English. These were recordings of four different counsellor-client interactions in generic (no specific intervention used) counselling sessions. After each video, participants were asked to identify and discuss the counselling skills and techniques used in the role plays. Prompts such as ‘What counselling skills did you observe being used in this video?’, and ‘What skills or techniques could or should have been used in this roleplay but were not evident?’ All items generated by the focus group discussions were listed on a whiteboard and confirmed by the group. Focus groups were audio recorded to ensure that no items were excluded. Lists from both focus groups were then combined into one spreadsheet.

### Step 2: item relevance

The factors generated in the first phases of the original ENACT's development were added to the items generated by the focus groups in Step 1 of this adaptation. Eight additional clinical psychologists then rated the complete list of items. All were registered clinical psychologists, with a mean of 17 years' experience (range: 8–28 years). All had at least two years of experience in public service, while five were employed in public sector positions at the time. All were active and practicing psychologists. Following the procedure used in the original tool development, (Kohrt *et al*., 2015b), the specialists who participated in this phase were asked to rate each item or process on a spreadsheet, first, according to how comprehensible the concept is to them and, second, how important they consider the concept or process to be for the purposes of counselling. For comprehensibility, items were rated 1 for ‘not very clear’; 2 for ‘generally clear’; and 3 for ‘very clear’. The importance of concepts or processes for good counselling was similarly ranked: 1 for ‘not usually essential’; 2 for ‘sometimes important’; and 3 for ‘important for all clients’. Means were calculated for the comprehensibility and importance scores of each item.

### Step 3: item utility and scoring

The goal of this step was to pilot the adapted tool in order to evaluate both the items and the instrument as a whole for face validity (participants' assessments of the importance of the items for effective psychotherapy), feasibility (participants' assessments of the observability of the behaviour, as well as user-friendliness of the tool's scoring procedures), and reliability (consensus among raters regarding their understanding of the items). These were assessed qualitatively through pilot testing and semi-structured discussions with four clinical psychologists who served as raters. All four were registered clinical psychologists working in public sector positions, with an average of 17 years of clinical experience (range: 7–26 years). The raters were asked to pilot the tool by rating eight audio recordings of real counselling sessions from Project MIND, where non-specialist counsellors delivered a 4-session blended motivational interviewing problem-solving intervention for patients living with diabetes or HIV (Myers *et al*., 2018b). Each rater was then interviewed to obtain feedback about the tool. Raters were asked to respond to questions such as, ‘Which items were unclear or difficult to rate?’, ‘Which items overlapped with one another?’, and ‘What was your overall experience of using the tool?’ Their responses were used to amend the tool further.

### Step 4: psychometric properties

To establish the internal consistency and reliability of the tool, two pairs of clinical psychologists and two pairs of registered psychological counsellors were each asked to rate 25 audio recordings of real counselling sessions delivered by non-specialist counsellors from Project MIND, for a total of 100 rating pairs. Cronbach's alpha was used to estimate the tool's internal consistency (reliability), while the intraclass correlation co-efficient, two-way random consistency model or ICC using average measures was used to measure inter-rater reliability.

## Results

### Step 1: item generation

Several themes emerged during the focus group discussions conducted in English to generate items for the adapted tool. One significant concern centred on counsellors' overreliance on intervention materials. Participants agreed that this represented a major barrier to the counsellor's ability to demonstrate essential competencies including the ability to establish rapport, actively listen to the patient, and foster a collaborative approach to the intervention. Another important theme was the importance of sensitivity to socio-economic status, race, gender, as well as the experience of trauma, especially given South Africa's painful socio-political history. By extension, participants discussed the importance of counsellors' ‘groundedness’ or the confidence to listen to patients relay difficult information or painful experiences without becoming judgmental, overwhelmed, or shocked. In sum, 98 items were generated by the focus group discussions.

### Step 2: item relevance

Along with the 115 items that were generated by the original study (Kohrt *et al*., 2015a), the 98 additional items generated during Step 1 yielded a complete list of 213 items. Nine duplicate items were identified and deleted. The remaining 204 items were then rated for comprehensibility and importance by eight clinical psychologists who had not participated in the previous step. The mean item importance score was 2.41 across all items, while the comprehensibility score was 2.71. The highest scoring items included empathy, warmth and non-verbal communication, while the lowest scoring factors included consistency of using only one technique, use of persuasion, and tension reduction. Scores of less than 2.5 represented items that were scored less than 3 by more than half of the raters. Consequently, items that had an average importance score of less than 2.5 were deleted from the list, leaving 96 items (Kohrt *et al*., 2015a). Guided by the original ENACT tool, the remaining 96 items were thematically coded according to their relationship to one another and the broader competency domains they were associated with. A total of 6 item domains were identified, with 19 items that were developed for the adapted measure.

### Step 3: item utility and scoring

Responses from the four specialists who piloted the 19-item ENACT-SA informed several important adaptations. Concerning the scoring system, the raters noted that the behaviours or skills on several items were not observed because there had been no opportunity to do so, and not because they were poorly performed. Consequently, a score of '0', denoting ‘not assessed’ was included in the tool's scoring system to accommodate instances when the opportunity to assess behaviours or skills did not emerge, but not necessarily through lack of competence.

Additional adaptations included the deletion of seven items on the ENACT-SA and the modification of an eighth. Three items were removed because they were considered specific to a particular modality. To retain the tool's focus on common therapeutic factors, a further three items concerning the assessment of the patient's life history, risk factors, and goal setting were deleted. Consequently, two domains were excluded (‘assessment’, and ‘boundaries and structure’). One item that was deemed difficult to observe, although essential to counsellor competence, was ‘Self-reflexivity: capacity for self-awareness and self-monitoring’. This item was excluded from the tool and incorporated into the tool's training guide.

For the final adapted measure, four competency domains were identified (communication; emotional engagement; process and intervention; and counsellor qualities and characteristics), comprised of a total of 12 items, each with four scoring possibilities (see Table 1). Several wording edits and clarifications were made to make the tool more accessible and user friendly to NSHWs.

**Table 1.** ENhancing Assessment of Common Therapeutic factors (ENACT) – SA

*Communication*
ITEM 1. VERBAL COMMUNICATION: REFLECTING, SUMMARISING, PARAPHRASING, CLARIFYING, AND USING OPEN-ENDED-QUESTIONS
◯ 0 NOT ASSESSED = this factor was not assessed because it was not applicable or because it did not emerge in the session.
◯ 1 NEEDS IMPROVEMENT = counsellor does not adequately facilitate the client's communication and expression; by failing to use skills such as reflecting, summarising, paraphrasing or clarifying, and mostly uses ‘yes/no’ questions, e.g., ‘Will you? Can you?’
◯ 2 DONE PARTIALLY = counsellor uses some skills to facilitate the client's communication, e.g. the counsellor uses open-ended questions but does not explore topics further or offer summaries for client reflection.
◯ 3 DONE WELL = counsellor uses open-ended questions, summarising, paraphrasing and clarifies statements, e.g., ‘What happened? Tell me more.’ or ‘If I understand correctly, it sounds as though…’
ITEM 2. COMMUNICATION DELIVERY: VOCAL TONE AND VOLUME, BODY LANGUAGE, GESTURES AND EYE CONTACT *(FOR REVIEW OF AUDIO RECORDED MATERIAL, FOCUS ON VOCAL TONE AND VOLUME; NON-LEXICAL GESTURES)
◯ 0 NOT ASSESSED = this factor was not assessed because it was not applicable or because it did not emerge in the session.
◯ 1 NEEDS IMPROVEMENT = counsellor does not make any eye contact or stares at client; and/or keeps arms crossed or turns away from client; and/or repeatedly interrupts patient; and/or talks too loudly or softly; and/or does not use ‘uh-huh’, ‘hmm’ or other culturally appropriate non-lexical gestures to signal interest.
◯ 2 DONE PARTIALLY = counsellor does not consistently use body language to express interest; and/or rarely makes eye contact; and/or shows limited emotion; and/or does not consistently use appropriate vocal tone/volume and/or uh-huh’, ‘hmm’ or other culturally appropriate non-lexical gestures to signal interest.
◯ 3 DONE WELL = makes appropriate eye contact throughout interaction; smiles when appropriate; sits at appropriate angle from patient and leans in to show interest; use of ‘uh-huh’, ‘hmm’ or other culturally appropriate non-lexical utterances to signal interest; adjusts vocal tone and volume as necessary.
ITEM 3. LISTENING AND ATTENDING SKILLS: ACTIVE LISTENING, ATTUNEMENT, TRACKING AND MIRRORING
◯ 0 NOT ASSESSED = this factor was not assessed because it was not applicable or because it did not emerge in the session.
◯ 1 NEEDS IMPROVEMENT = counsellor is not paying attention or listening to what the client is saying, e.g. the counsellor's asks for information that the client has already given; and/or the counsellor frequently loses track of the client's story.
◯ 2 DONE PARTIALLY = counsellor occasionally loses track of the client's story and is not sufficiently attuned to the client; e.g. the counsellor does not follow up on important and relevant information provided by the client.
◯ 3 DONE WELL = counsellor demonstrates attentiveness and attunement to the client, by listening actively, mirroring the client and tracking the client's story; e.g. the counsellor asks follow-up questions that demonstrate that the counsellor is attending to the client's story.

*Emotional Engagement*
ITEM 4. DEMONSTRATION OF WARMTH, EMPATHY, GENUINENESS AND RESPECT
◯ 0 NOT ASSESSED = this factor was not assessed because it was not applicable or because it did not emerge in the session.
◯ 1 NEEDS IMPROVEMENT = counsellor is critical, disrespectful, hostile, or dismissive of client's concerns or complaints; and/or seems artificial or insincere.
◯ 2 DONE PARTIALLY = counsellor is generally warm and friendly to the client but does not demonstrate the ability to put him/herself in the experience of the client; and/or is respectful but aloof or insincere.
◯ 3 DONE WELL = counsellor demonstrates that he/she understands the experience of the client in a warm, genuine and sincere manner, and is respectful of the client.
ITEM 5. RESPONSIVENESS TO CLIENT: CONTAINING, AFFIRMING, ACKNOWLEDGING, PRAISING AND GIVING FEEDBACK
◯ 0 NOT ASSESSED = this factor was not assessed because it was not applicable or because it did not emerge in the session.
◯ 1 NEEDS IMPROVEMENT = counsellor ignores client's distress, and/or doesn't acknowledge client's communication by affirming and/ or giving feedback.
◯ 2 DONE PARTIALLY = counsellor is generally responsive to the client but does not adequately contain the client; and/or does not affirm or give feedback.
◯ 3 DONE WELL = counsellor is responsive to the client by being present and engaged, containing and affirming, praising and giving feedback when appropriate.
ITEM 6. ABILITY TO BUILD RAPPORT
◯ 0 NOT ASSESSED = this factor was not assessed because it was not applicable or because it did not emerge in the session.
◯ 1 NEEDS IMPROVEMENT = counsellor does not introduce him/herself or attempt to make the patient feel comfortable and/or the counsellor dominates the session talking about his/her own experiences.
◯ 2 DONE PARTIALLY = counsellor introduces him/herself but does not make an effort to make the client feel comfortable.
◯ 3 DONE WELL = counsellor introduces him/herself, tries to make the client feel comfortable, attended to, and that his/her agenda is prioritised.
ITEM 7. USING A CLIENT-CENTRED COLLABORATIVE APPROACH
◯ 0 NOT ASSESSED = this factor was not assessed because it was not applicable or because it did not emerge in the session.
◯ 1 NEEDS IMPROVEMENT = counsellor is overly directive and prescriptive; instructing and lecturing the client.
◯ 2 DONE PARTIALLY = counsellor checks in with the client to ensure understanding but does not adjust to client's corrections, and/or does not allow for sufficient collaboration.
◯ 3 DONE WELL = counsellor seeks out client's explanation for difficulties; checks in with the client to ensure understanding of each other, and being receptive to corrections; and, creates space for the client to ask questions.
*Process and Intervention*
ITEM 8. MANAGING CONFIDENTIALITY
◯ 0 NOT ASSESSED = this factor was not assessed because it was not applicable or because it did not emerge in the session.
◯ 1 NEEDS IMPROVEMENT = counsellor does not address confidentiality and/or breaches confidentiality, e.g. by telling the client something about another client.
◯ 2 DONE PARTIALLY = counsellor tells the client that everything is confidential without explaining exceptions such as harm to self or others, or the counsellor states that everything is confidential while conducting the session in a setting/location that is not private and information may not be kept confidential, e.g. a waiting room where other people are present.
◯ 3 DONE WELL = counsellor explains that all client-counsellor discussions are confidential with the exception of harm to self and others, and the clinician adjusts conversation topics based on the amount of privacy the setting/location affords.
ITEM 9. IDENTIFYING EXTERNAL RESOURCES
◯ 0 NOT ASSESSED = this factor was not assessed because it was not applicable or because it did not emerge in the session.
◯ 1 NEEDS IMPROVEMENT = counsellor does not explore the client's access to other useful resources, including the client's secure relationships.
◯ 2 DONE PARTIALLY = counsellor asks about the client's access to resources but does not incorporate them into the counselling strategy.
◯ 3 DONE WELL = counsellor explores and lists all the resources that the client has access to, including secure relationships, and works with the client to incorporate them into the counselling plan.
*Counsellor Qualities and Characteristics*
ITEM 10. FLEXIBILITY: ABILITY TO ADAPT TO CLIENT
◯ 0 NOT ASSESSED = this factor was not assessed because it was not applicable or because it did not emerge in the session.
◯ 1 NEEDS IMPROVEMENT = the counsellor is unfamiliar with the intervention or has not internalised the intervention materials and principles sufficiently and so is over-reliant on these to direct the interaction, e.g. by reading directly from the manual for more or most of the session.
◯ 2 DONE PARTIALLY = counsellor is partially able to adapt to the client but seems unfamiliar with the intervention materials and principles, which impedes the counsellor's ability to adapt to the client's needs.
◯ 3 DONE WELL = counsellor is familiar with the intervention materials and principles and so is able to adapt to the client's needs within the framework of the intervention.
ITEM 11. APPROPRIATE SELF-CONFIDENCE AND GROUNDEDNESS
◯ 0 NOT ASSESSED = this factor was not assessed because it was not applicable or because it did not emerge in the session.
◯ 1 NEEDS IMPROVEMENT = counsellor is clearly anxious or uncomfortable and responds to the client's story with alarm or shock.
◯ 2 DONE PARTIALLY = counsellor is somewhat anxious or occasionally appears unsure of him/herself.
◯ 3 DONE WELL = counsellor is appropriately relaxed, calm, comfortable and appropriately confident.
ITEM 12. SENSITIVITY TO RACE, SES, CULTURE, GENDER, AND TRAUMA
◯ 0 NOT ASSESSED = this factor was not assessed because it was not applicable or because it did not emerge in the session.
◯ 1 NEEDS IMPROVEMENT = counsellor demonstrates some form of prejudice (e.g. sexism, racism); and/or is insensitive about the trauma that the client has experienced; e.g. the counsellor forces the client to discuss trauma and share details before assessing readiness/willingness to do trauma work.
◯ 2 DONE PARTIALLY = counsellor is generally sensitive and open-minded but is occasionally harsh or dismissive of the client's experiences of trauma or prejudice; e.g. the counsellor makes comments that downplay or invalidate the client's experiences, such as ‘it's not so bad’.
◯ 3 DONE WELL = counsellor is sensitive to socio-cultural issues and the roles these might play in the client's life; and, the counsellor is gentle with and sensitive to the client's experience of trauma.

### Step 4: psychometric properties

The average item ICC (2, 4) for the clinical psychologist raters, based on ratings of 25 audio recordings of counselling sessions per pair, was 0.66 (95% CI 0.60–0.71). The ICC for the counselling psychologist raters, based on ratings of the same audio recordings, was 0.69. Cronbach's alpha, based on 25 clinical psychologist ratings of counselling session recordings was 0.86. Cronbach's alpha for 25 counselling psychologist ratings of recordings was 0.88. When exploring the ICCs separately by domain, the ICCs were relatively consistent.

## Discussion

In this study, the original ENACT rating scale, developed to facilitate the assessment of NSHW counselling competence, was adapted for the South African context. Unlike previous studies that adapted the original ENACT tool by modifying existing items (Kohrt *et al*., 2020; Asher *et al*., 2021), this study ‘started from scratch’ by including ratings by local mental health professionals of the original pool of total items as well as local development of additional items. The findings show that 9 (50% of the original items) were nearly exact to the original ENACT; a number of items were deemed less relevant (family involvement, suicide screening, patient-centred explanatory models), and new items focused on counsellor qualities (flexible, grounded, culturally sensitive), were considered more relevant for rating observable competencies. Therefore, this study has uniquely added value in converging on some common factors, as well as identifying contextual, programmatic, and cultural differences.

Of the 18 original ENACT items, nine items were retained in modified form in the ENACT-SA. Items that promoted rapport-building, empathy, warmth, non-judgment, collaboration, active listening, as well as good non-verbal and verbal communication skills, were nearly exact to the original ENACT. These interpersonal skills perhaps represent a core set of competencies that might be relevant for any context and is consistent with the findings of studies of common therapeutic factors (Wampold, 2015; Finsrud *et al*., 2022). With research suggesting that interpersonal processes are strongly associated with treatment outcomes for non-specialist delivered psychological treatments in LMIC (Singla *et al*., 2017), maximising the presence of these therapist factors will arguably optimise the effectiveness of any intervention. Previous South African studies have noted that both patients and health providers consider these competencies as critical for promoting patient uptake and engagement in task-shared therapies (Myers *et al*., 2018a; Sorsdahl *et al*., 2021).

Several items of the original ENACT were deemed more variable and less relevant to the South African context and were better and more flexibly addressed by other items, specifically, family involvement, suicide screening, and patient-centred explanatory models. Other cultures may place more emphasis on family involvement in mental health and psychosocial services, requiring different items to assess the competence of including family members. This was the case original ENACT where experiences in Nepal, Uganda, and Liberia found that family members were often included in sessions (Kohrt *et al*., 2018; Jordans *et al*., 2021; Leichner *et al*., 2021). In South Africa, the benefits of family involvement are not a given across all cultures. Consequently, the decision to include family members in the intervention was thought better determined by factors such as cultural sensitivity, identifying external resources, and flexibility. Other differences in context were that non-specialists were the only health workers available to screen for suicidality at the original pilot sites for ENACT, and therefore, self-harm assessment and safety planning were seen as an essential responsibility of these cadres. In the South African context, referral protocols for suicidal behaviour are generally available, and suicide screening was deemed to form part of an assessment process. Where suicidality emerges within a counselling session, in concert with formal protocols and institutional mechanisms, the appropriate response would be subsumed within factors such as identifying external resources. Factors such as sensitivity to culture and using a collaborative approach were deemed more broadly applicable and could encompass explanatory models. These differences highlight the need for adapting the ENACT to other cultures and contexts is an important undertaking, especially given the critique of western psychological constructs and implementation strategies being imposed on other cultures.

Further, original ENACT items that were associated with particular intervention modalities were excluded from the SA-ENACT. For example, the item ‘Coping strategies and new behaviours’ intended to measure counsellors' abilities to identify and implement healthy new coping mechanisms and behaviours was considered treatment-specific and likely determined by the kind of intervention employed. Treatment-specific competencies (also described as ‘intervention fidelity’ or ‘intervention integrity’) refer to the degree to which the counsellor delivers the content of the programme as specified in the manual to the target person or group. It is recommended that the ENACT-SA be used in conjunction with a measure of treatment-specific competencies (Pedersen *et al*., 2021), and have been used in several projects in South Africa including CoBALT (Fairall *et al*., 2018) and Project MIND (Myers *et al*., 2018b). Similarly, while factors related to the assessment of patients were rated highly and included in the piloted version of the tool, they were excluded from the final version. This was done under the assumption that assessment procedures, including baseline questionnaires or other history-taking and assessment protocols, should routinely be in place at any intake. In this way, a clearer emphasis on the tool's evaluation of therapeutic factors could be retained.

Three unique competencies that focused on counsellor qualities (flexible, grounded, culturally sensitive), were generated through the South Africa adaptation process and deemed more relevant for rating observable competencies. First, ‘Flexibility: ability to adapt and cope’, was highlighted by specialists involved in the first and third steps of the tool's adaptation process. This was a result of the ways in which counsellors' over-reliance on intervention materials and scripts became obstacles to building therapeutic rapport and using a collaborative approach. Feedback from specialists involved in this step noted that the adapted tool did not adequately address this issue. They observed that inadequate knowledge and internalisation of the intervention principles, procedures and materials were evident in counsellors' lack of flexibility and inability to adapt to patients' needs. This item was reworded to incorporate a clearer focus on assessing counsellors' knowledge of the intervention. This adaptation also created the opportunity for trainers to ensure that counsellors' understanding and familiarity with the intervention is adequate.

Second, ‘appropriate self-confidence and groundedness’ was considered a key characteristic of a counsellor. The psychotherapeutic literature has emphasised the importance of providing a ‘holding’ relationship for the service user; this requires that the counsellor is able to convey a certain degree of assuredness about the process that therapist and client are embarking on, as well as a real understanding of and experience with the issues faced by the user. Third, sensitivity to race, SES, gender, and trauma was also considered an important quality of a counsellor. Given the complex nature and diversity of cultural identities, language, and various social determinants sensitivity to these issues is paramount. This inclusion is consistent with the recently developed child version of the ENACT – the WeACT – that includes an item similar to Item #12 based on the recognised importance in a multicultural context (Jordans *et al*., 2021). Overall, this study has unique added value in converging on some common factors, as well as identifying contextual, programmatic, and cultural differences.

Another important adaptation was the inclusion of a zero score for factors that were not observed, which was needed because the focus of rating was actual sessions rather than standardised role plays which can be designed to include all competencies of interest (Ottman *et al*., 2020). For example, in sessions with clients, the raters reported that counsellors did not always explicitly refer to the management of confidentiality. This was not due to a lack of skill or competence, but because the session was a second or third one, when it was reasonable to assume that matters concerning confidentiality might have been addressed in the first session. As such, marking ‘needs improvement’ (a score of 1), seemed inaccurate. In order to assess this more carefully, ratings of all sessions would be appropriate, albeit time consuming.

Finally, the adapted tool's internal consistency reliability was good, while the inter-rater reliability was verging on acceptable. When exploring variation by domain there was little variation. Given that we used ‘real’ sessions rather than standard role plays for establishing ICC, this may have led to an artificially low ICC of scores, particularly given the high Cronbach's alpha reported. Role play sessions are typically prepared and much ‘neater’ than real-life interactions as you can ensure all competencies are addressed in the role play. This would remove the option of not-applicable (NA) and result in an increased ICC. Importantly, there were no discernible differences in the ICCs or internal consistency coefficients between a clinical psychologist and registered counsellor raters. This finding supports claims that supervision of NSHW can be successfully task-shared without necessarily sacrificing quality (Jacobs *et al*., 2020).

Study limitations need consideration when interpreting these findings. First, we were not able to measure years of exposure or experience among study participants and this may have had an influence on item selection and rating. Second, given the moderate ICCs found in the current study, future studies should consider evaluating inter-rater reliability with standardised role plays that can maximise within-session variance and eliminate potential biases in rating due to judgements related to not-applicable. Third, not taking into consideration the perceptions and experiences of the recipient of the EBI of the identified ENACT-SA items may be considered a limitation. However, the overriding aim of the ENACT is to provide a means of transferring specialist skills and expertise to non-specialists. Given that the EBIs are derived from psychotherapies, the specialist training and experience of experienced psychologists and practising psychotherapists were deemed the most valuable source of expertise in this respect. Finally, an assessment of the ENACT-SA tool's translation into other South African official languages would test its utility and face validity for future trials of task-sharing interventions.

Despite these limitations, the findings of this study contribute substantially to counsellor quality competencies in low-resourced settings. First, the process of adapting the ENACT to South Africa provides a framework for other countries wishing to adapt the measure to their context. Second, the adaptations have rendered a tool that is likely to be widely applicable to a broad range of settings, and will be of utility to the training, monitoring, and supervision of a wider range of health professionals in other contexts. It is recommended that this tool be used in conjunction with assessment protocols and treatment-specific competency and treatment fidelity to fully assess implementation and potential mechanisms of therapeutic change. Third, by replicating the original ENACT development process, we were able to demonstrate the relevance, feasibility, and high clinical utility of 9 of the original ENACT items in a novel cultural context. Expanding on this, we demonstrated that it is feasible to measure counsellor qualities, such as flexibility, groundedness, and cultural sensitivity, in the context of role plays and observed clinical encounters. This is critical because of its relevance in the context of diverse clients and programme beneficiaries. The development of counsellor quality competencies can now be evaluated in other settings, with the potential for inclusion in the EQUIP platform (https://equipcompetency.org/en-gb) version of ENACT, which is now being scaled up globally.

## Footnotes

† The notes appear after the main text.

1 To be registered as a psychologist with the Health Professions Council in South Africa, a minimum of a Masters degree in the relevant sub-discipline from an accredited tertiary institution is required.
